# bvnGPS: a generalizable diagnostic model for acute bacterial and viral infection using integrative host transcriptomics and pretrained neural networks

**DOI:** 10.1093/bioinformatics/btad109

**Published:** 2023-03-01

**Authors:** Qizhi Li, Xubin Zheng, Jize Xie, Ran Wang, Mengyao Li, Man-Hon Wong, Kwong-Sak Leung, Shuai Li, Qingshan Geng, Lixin Cheng

**Affiliations:** Shenzhen People’s Hospital, First Affiliated Hospital of Southern University of Science and Technology, Second Clinical Medicine College of Jinan University, Shenzhen 518020, China; John Hopcroft Center for Computer Science, Shanghai Jiao Tong University, Shanghai, China; Shenzhen People’s Hospital, First Affiliated Hospital of Southern University of Science and Technology, Second Clinical Medicine College of Jinan University, Shenzhen 518020, China; Department of Computer Science and Engineering, The Chinese University of Hong Kong, Shatin, New Territories, Hong Kong; Great Bay University, Dongguan, China; Shenzhen People’s Hospital, First Affiliated Hospital of Southern University of Science and Technology, Second Clinical Medicine College of Jinan University, Shenzhen 518020, China; John Hopcroft Center for Computer Science, Shanghai Jiao Tong University, Shanghai, China; Department of Computer Science and Engineering, The Chinese University of Hong Kong, Shatin, New Territories, Hong Kong; Shenzhen People’s Hospital, First Affiliated Hospital of Southern University of Science and Technology, Second Clinical Medicine College of Jinan University, Shenzhen 518020, China; Department of Computer Science and Engineering, The Chinese University of Hong Kong, Shatin, New Territories, Hong Kong; Department of Computer Science and Engineering, The Chinese University of Hong Kong, Shatin, New Territories, Hong Kong; Department of Applied Data Science, Hong Kong Shue Yan University, North Point, Hong Kong; John Hopcroft Center for Computer Science, Shanghai Jiao Tong University, Shanghai, China; Shenzhen People’s Hospital, First Affiliated Hospital of Southern University of Science and Technology, Second Clinical Medicine College of Jinan University, Shenzhen 518020, China; Shenzhen People’s Hospital, First Affiliated Hospital of Southern University of Science and Technology, Second Clinical Medicine College of Jinan University, Shenzhen 518020, China

## Abstract

**Motivation:**

The confusion of acute inflammation infected by virus and bacteria or noninfectious inflammation will lead to missing the best therapy occasion resulting in poor prognoses. The diagnostic model based on host gene expression has been widely used to diagnose acute infections, but the clinical usage was hindered by the capability across different samples and cohorts due to the small sample size for signature training and discovery.

**Results:**

Here, we construct a large-scale dataset integrating multiple host transcriptomic data and analyze it using a sophisticated strategy which removes batch effect and extracts the common information from different cohorts based on the relative expression alteration of gene pairs. We assemble 2680 samples across 16 cohorts and separately build gene pair signature (GPS) for bacterial, viral, and noninfected patients. The three GPSs are further assembled into an antibiotic decision model (bacterial–viral–noninfected GPS, bvnGPS) using multiclass neural networks, which is able to determine whether a patient is bacterial infected, viral infected, or noninfected. bvnGPS can distinguish bacterial infection with area under the receiver operating characteristic curve (AUC) of 0.953 (95% confidence interval, 0.948–0.958) and viral infection with AUC of 0.956 (0.951–0.961) in the test set (*N* = 760). In the validation set (*N* = 147), bvnGPS also shows strong performance by attaining an AUC of 0.988 (0.978–0.998) on bacterial-versus-other and an AUC of 0.994 (0.984–1.000) on viral-versus-other. bvnGPS has the potential to be used in clinical practice and the proposed procedure provides insight into data integration, feature selection and multiclass classification for host transcriptomics data.

**Availability and implementation:**

The codes implementing bvnGPS are available at https://github.com/Ritchiegit/bvnGPS. The construction of iPAGE algorithm and the training of neural network was conducted on Python 3.7 with Scikit-learn 0.24.1 and PyTorch 1.7. The visualization of the results was implemented on R 4.2, Python 3.7, and Matplotlib 3.3.4.

## 1 Introduction

Accurate diagnosis of bacterial and viral infections can assist clinicians in deciding treatment strategies and the confusion leads to missing the best therapy occasion and results in poor prognoses (Ferrer et al. 2014). Clinically, broad-spectrum antibiotics can cover most of the bacterial infections and a broad-spectrum antiviral is effective against a wide range of viruses. However, bacterial and viral infections cause similar clinical manifestations and the confusion of them leads to misdiagnosis and overuse of antibiotics. The abuse of antibiotics increases costs and causes antimicrobial resistance (Fridkin et al. 2014), which is a significant threat to human health. Microbiological culture is the mainstream method for identifying the pathogens of infection, but it has a long culture cycle and high false negatives (Towns et al. 2010). Additionally, in some cases, it is difficult to extract the infected tissues directly for pathogen detection. Hence, an operable and early detection method to distinguish viral and bacterial infections is of vital importance.

The host’s immune response to infection provides a noninvasive diagnostic strategy by detecting blood gene expression (Pankla et al. 2009; Almansa et al. 2012, 2015; Scicluna et al. 2015; Burnham et al. 2017). In recent years, host gene expression has been studied as an indicator for pathogen diagnostics with the advantage of rapid detection by real-time polymerase chain reaction (McHugh et al. 2015; Scicluna et al. 2015; Cheng et al. 2018; Sweeney et al. 2018; Cheng et al. 2020; Liu et al. 2021). However, it remains a significant challenge in lack of high accuracy for these machine learning and statistical methods (Sweeney et al. 2018; Li et al. 2022), which is partially caused by insufficient training data. Importantly, the lack of data in infection classification is not accused of the shortage of patients, but that the cohorts from different assays and platforms cannot be appropriately combined to create an adequate training set because of the batch effect (Johnson et al. 2007; Liu et al. 2019).

Previously, we proposed an algorithm named individualized Pair Analysis of Gene Expression (iPAGE) to extract the common information from different cohorts, which utilizes the relative expression changes of gene pairs to retain the most reliable gene information and ensure robustness (Zheng et al. 2021; Wang et al. 2022; Wu et al. 2022). iPAGE is effective in addressing the problem of data normalization and batch effect removal (see Section 2), which serves as a sophisticated way in integrating gene expression cohorts to enlarge the sample size of diseases, such as sepsis (Liu et al. 2020; Zheng et al. 2021) and tumors (Wu et al. 2022; Cheng et al. 2023). By augmenting the training data, it enables the use of deep neural networks for model construction, which usually achieves state-of-the-art accuracy in bioinformatic applications.

**Figure 1:**
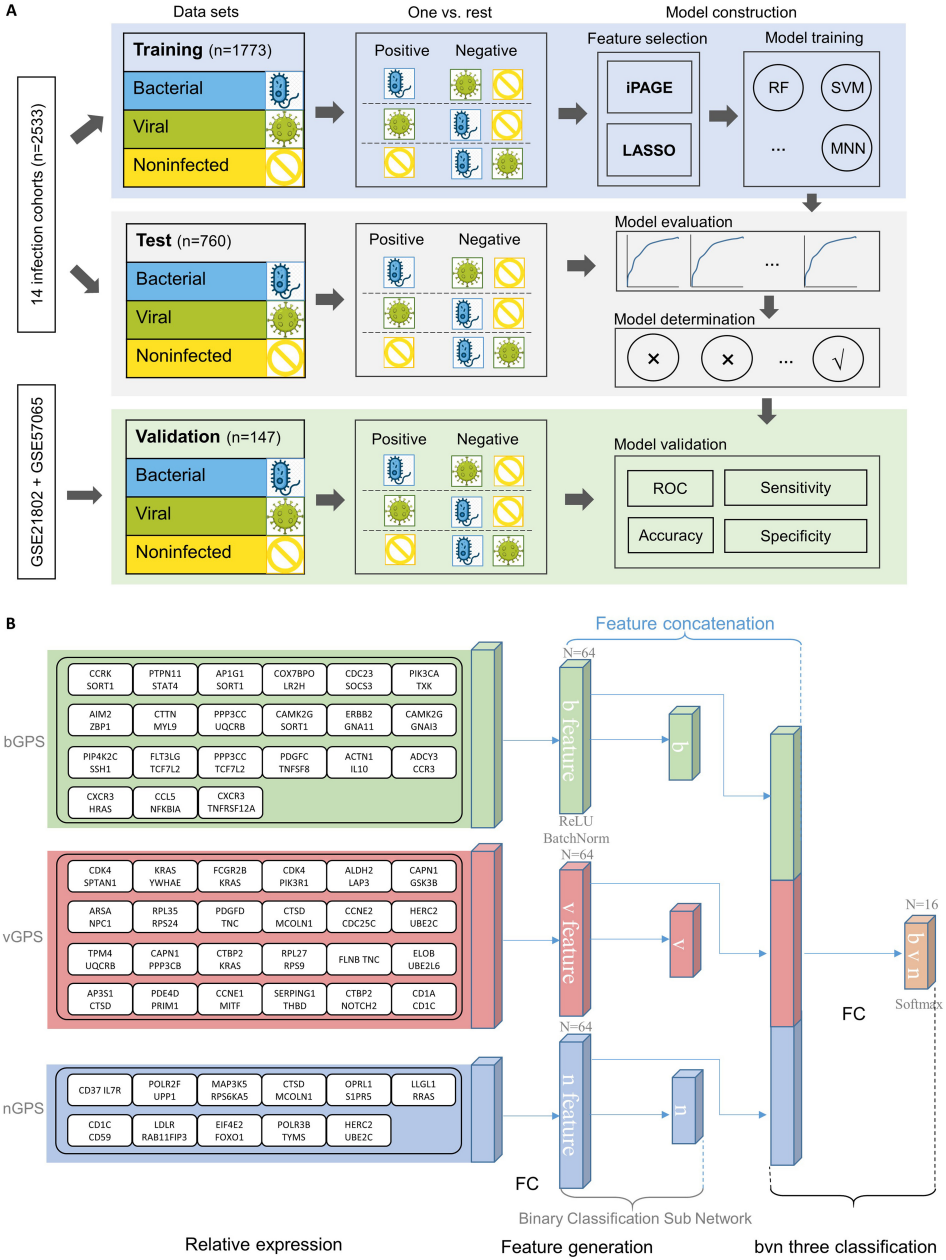
An unbiased procedure for the construction, evaluation, determination, and validation of the antibiotic decision model. (A) For each one-versus-other classification, gene pairs were filtered out using iPAGE and then gene pair signatures (GPSs) were built using LASSO from the training set. After that, four models were trained to assemble the GPSs of each category. The optimal one with the highest AUC was evaluated using an external validation set. See Section 2 for details. (B) The structure and hyperparameters of bvnGPS

In this study, to build a diagnostic model with high accuracy in discriminating viral and bacterial infection, we used iPAGE to integrate data from different cohorts and boost the sample size for model training (Fig. 1A). Then, we separately extracted the gene pair signatures (GPSs) for viral infection, bacterial infection, and noninfected patients. After that, an antibiotics decision model named bacterial–viral–noninfected GPS (bvnGPS) was built based on the three GPSs using multiclass neural networks to determine the infection type of a patient (Fig. 1B). Finally, we evaluated the performance of bvnGPS in an internal test set and an external validation set.

## 2 Materials and methods

### 2.1 Establishment of datasets

We performed a systematic search for cohorts from the Gene Expression Omnibus (GEO) (Edgar et al. 2002) database satisfying the following inclusion criteria: (i) cohorts with bacterial infections or viral infections; (ii) cohorts with hospitalization and clinical information; and (iii) cohorts with whole blood samples. Samples were excluded due to (i) sarcoid and cancer; (ii) unknown pathogens; and (iii) coinfection with bacteria and virus (Supplementary Fig. S1). From the 3203 samples across 16 cohorts, we filtered 2680 samples for subsequent analysis (Supplementary Fig. S1 and Table 1). Patients with *Escherichia coli*, methicillin-resistant *Staphylococcus aureus*, tuberculosis, influenza A virus subtype H1N1 etc. were included in the current study to identify bacterial infection and viral infection.

**Table 1.** Datasets used in the study.

Dataset accession	Number of sample	Organs	Platform	Cohort details	Pathogen	Control	Bacteria	Virus
Training and test set								
GSE20346	81	Whole blood	GPL6947	Adult	Unknown bacterial pneumonia; influenza	36	26	19
GSE40012	176	Whole blood	GPL6947	Adult	Unknown bacterial pneumonia; influenza; SIRS	36	61	79
GSE40396	65	Whole blood	GPL10558	Children	Bacteria; adenovirus; enterovirus; rhinovirus; HHV6	22	8	35
GSE42026	92	Whole blood	GPL6947	Children	*Streptococcus*; *Staphylococcus*; influenza; RSV	33	18	41
GSE66099	95	Whole blood	GPL570	Children	Bacteria; influenza; HSV; CMV; BK; adenovirus	47	41	7
GSE60244	133	Whole blood	GPL10558	Adult	Gram-positive and atypical; influenza; RSV; MPV	40	22	71
GSE27131	21	Whole blood	GPL6244	Adult	H1N1	7	0	14
GSE111368	359	Whole blood	GPL10558	Adult	H1N1; H3N2; influenza	130	0	229
GSE28750	41	Whole blood	GPL570	Adult	Bacteria	20	21	0
GSE42834	208	Whole blood	GPL10558	NA	TB	143	65	0
GSE69528	138	Whole blood	GPL10558	NA	Septicemic melioidosis; other sepsis,	55	83	0
GSE63990	280	Whole blood	GPL571	Adult	Multiple bacteria; multiple virus	90	73	117
GSE68310	725	Whole blood	GPL10558	Adult	Influenza A/B virus; coronavirus; rhinovirus	0	0	725
GSE6269	119	Whole blood	GPL570, GPL2507	Both	Influenza A virus; *Escherichia coli*;	6	85	28
					*Staphylococcus aureus* and *Streptococcus pneumoniae*			
Total	2533					665	503	1365
Validation set								
GSE57065	107	Whole blood	GPL570	Adult	Bacteria	25	82	0
GSE21802	40	Whole blood	GPL6102	Adult	H1N1	4	0	36
Total	147					29	82	36

SIRS, systemic inflammatory response syndrome; HHV6, human herpesvirus 6; RSV, respiratory syncytial virus; HSV, herpes simplex virus; CMV, cytomegalovirus; BK, BK virus; MPV, human metapneumovirus; H1N1, influenza A virus subtype H1N1; H3N2, influenza A virus subtype H3N2; TB, tuberculosis; NA, unknown.

The samples of 14 cohorts in the discovery set were divided into 70% (1876) for training and 30% (804) for testing (Table 1). The training set is applied to extract biomarkers and further train classifiers while the test set is employed to evaluate the performance and determine the hyperparameters of bvnGPS. To verify the generalization ability of bvnGPS, 147 patients in GSE21802 and GSE57065 were used for external validation. To demonstrate the robustness and simplicity of the GPS procedure, no additional preprocessing was performed on the raw expression cohorts.

### 2.2 iPAGE

Our method extracted biomarkers from one-versus-other binary classification for each category in three tasks: bacterial-versus-other, viral-versus-other, and non-infected-versus-other.

####  

Individualized Pair Analysis of Gene Expression (iPAGE) was employed to screen out gene pairs as the biomarkers (Zheng et al. 2021; Li et al. 2022; Wang et al. 2022). Different cohorts have batch effects in widely different background measurements including amplification reagent used, extraction procedures, and platforms (Johnson et al. 2007). In accordance with previous researches, the absolute expression abundance of genes was highly influenced by the batch effect between cohorts (Heinäniemi et al. 2013; Wang et al. 2015; Cheng et al. 2016a,b; Liu et al. 2019). However, it is disastrous when cohorts were integrated using methods with inappropriate assumptions.

**Figure 2:**
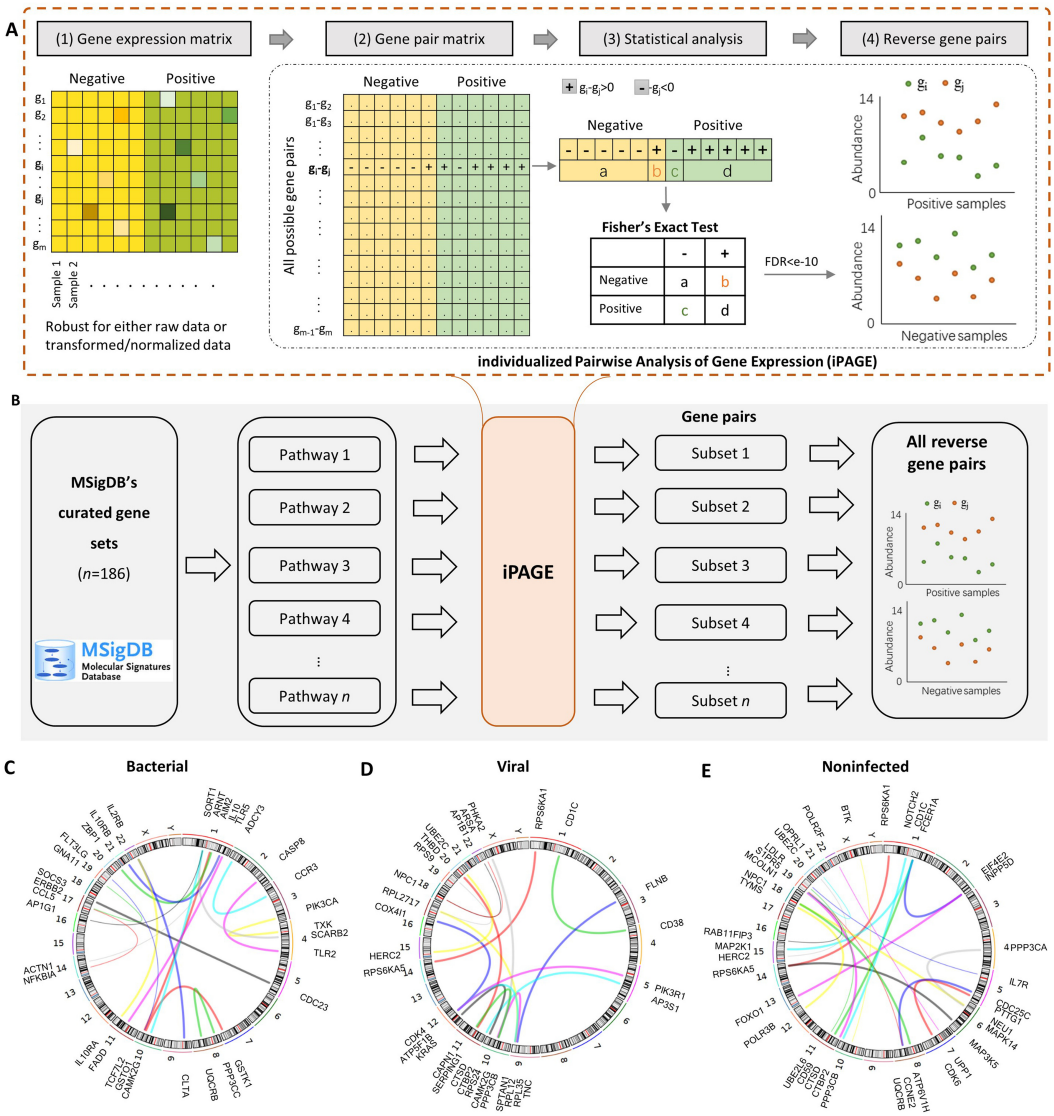
(A) The workflow of iPAGE. (1) The gene expression data including two groups of samples. (2) Transfer gene expression matrix to gene pair matrix. Relative expression between every gene pairs were calculated in each sample. (3) Statistical analysis across all samples. Gene pairs with Fisher’s exact test *P*-value <10^−16^ were kept for subsequent analysis. (4) Schematic of the expression of the identified gene pairs across the two groups of samples. See Section 2 for details. (B) Selecting reverse gene pairs using biological pathways. (C–E) Genome locations of the gene pairs for bGPS, vGPS, and nGPS, respectively

Generally, according to previous studies (Heinäniemi et al. 2013; Wang et al. 2015; Zheng et al. 2021), the relative expression between genes within a sample is reliable and foundational to detect the genetic differences between different groups. The relative expression facilitates more accurate detection with precise information. Thus, the relative expression changes were calculated between all pairs of genes (Fig. 2A). The pipeline of iPAGE is described as follows (Fig. 2A).

Formally, the k-th sample was detected with multi genes $\left(g_{1}^{\left(k\right)},g_{2}^{\left(k\right)},\ldots ,g_{m}^{\left(k\right)}\right)$. In the k-th sample, the raw expression intensity of gene on gene i is $g_{i}^{\left(k\right)}$, while the label $Y^{\left(k\right)}$ equals to 0 for negative and 1 for positive. The relative expression of gene pair $\left(g_{i}^{\left(k\right)},g_{j}^{\left(k\right)}\right)$ is defined as:


(1) $$r^{\left(k\right)}=I\left(g_{i}^{\left(k\right)}-g_{j}^{\left(k\right)}\right),$$

where Ix=1, if x>0-1, if x≤0. If $g_{i}^{\left(k\right)}$ is greater than $g_{j}^{\left(k\right)}$, the relative expression of $\left(g_{i}^{\left(k\right)},g_{j}^{\left(k\right)}\right)$ was 1, otherwise, it should be -1.

With $r_{ij}^{\left(k\right)}=1$ as $g_{i}^{\left(k\right)}>g_{j}^{\left(k\right)}$ and $r_{ij}^{\left(k\right)}=-1$ as $g_{i}^{\left(k\right)}\leq g_{j}^{\left(k\right)}$, contingency table was calculated as follows.

**Table:** 

	$g_{i}^{\left(k\right)}>g_{j}^{\left(k\right)}$	$g_{i}^{\left(k\right)}\leq g_{j}^{\left(k\right)}$
Negative	$\sum_{k=1}^{n}r_{ij}^{\left(k\right)}{(1-Y}^{\left(k\right)})$	$\sum_{k=1}^{n}{-r}_{ij}^{\left(k\right)}\times {(1-Y}^{\left(k\right)})$
Positive	$\sum_{k=1}^{n}r_{ij}^{\left(k\right)}\times Y^{\left(n\right)}$	$\sum_{k=1}^{n}{-r}_{ij}^{\left(k\right)}\times Y^{\left(n\right)}$

For shrinking the variance, Fisher’s exact test was used as a screen. The cutoff of Bonferroni corrected false discovery rate $10^{-10}$ was set to identify significant gene pairs with reverse expression change between the positive and negative groups. Before Fisher’s exact test, to extract biologically significant genes, pair extraction was performed within each pathway (Fig. 2B), owing to genes involved in the same pathway are generally assumed to be functionally related. Based on Molecular Signatures Database (MsigDB), 186 gene sets derived from the curated gene sets (C2)-Kyoto Encyclopedia of Genes and Genomes (KEGG) were separately used for pair selection using iPAGE.

### 2.3 Model construction and hyperparameter selection

Using LASSO, we selected the least number of pairs as GPSs for bacterial infection, viral infection, and noninfected, respectively. With the 54 unique gene pairs determined, the gene expression datasets were transferred to relative expression with (1). Then, machine learning models were employed to assemble the relative expression of these gene pairs for multiclass classification. In bvnGPS, the relative expressions are processed into bacterial, viral, and noninfected features, and then the generated features are used for the classification of infection. For the multiclass neural network, the model consists of a feature generation module and a bvn three classification module.

In the feature generation module, binary labels are used to train a single class of features, such as the bacterial feature parameters (green), viral feature parameters (red), and noninfected feature parameters (blue) (Fig. 1B). The feature generation module contains one hidden layer with Rectified Linear Unit (ReLU) and batchnorm. Concatenating different primary networks is a common technique for multitask neural network and extracting features from different angles. In this study, binary sub-task layers were used as primary layer to generate higher level features of bacterial, viral, noninfections because the numbers of gene pairs for different infection types are in different dimension. Using multitask neural network directly will cause unbalanced weights between bacterial, viral, and noninfections.

In the bvn three classification module, bvnGPS concatenates the features of bacteria, virus, and noninfected, and classifies them into three categories. bvnGPS contains multiply hidden layers with ReLU activation function and batchnorm, the three neurons in the final layer corresponding to bacterial, viral, and noninfected categories with softmax activation function.

We trained this model as follows: first, the parameters of the feature generation module were trained with the binary classification. Then, with the parameters of the feature generation module fixed, we train the bvn three classification module with infection labels in three categories. (Fig. 1B). Batchsize was fixed as 128, the learning rate was fixed as 0.001, the optimizer was Adam, and other parameters were selectively searched: (i) in the feature generation module, the numbers of perceptron in hidden layers were tuned in Almansa et al. (2015; 16, 32, 64, 128); and (ii) in the bvn three classification module, we searched the no hidden layer, one hidden layer, and two hidden layers. And the number of perceptron in each layer was searched from 2 to 128. For early stopping in the neural network, 20% of samples in the training set were partitioned with random seed 1.

For comparison, we employed the following models for three-categories classification: decision tree (DT), random forest (RF) with linear discriminant analysis (LDA), and support vector machines (SVM). For DT, the max depth of layers was set by default. For RF with LDA, the RF’s parameters were in the default setting and the component number in LDA remained as 1. For SVM, we fixed the decision function as one-versus-one to vote and observed the search of hyperparameter for SVM was performed with the penalty term from 0.1 to 2. Weights and parameters of models were fixed after being modified on the training set without any subsequent modification.

### 2.4 Performance evaluation

To compare the performance of the multiclass model, area under the receiver operating characteristic curve (AUC) was employed to assess the classification ability of models to discriminate the positive samples from the negative ones. Since it has more excellent medical value to distinguish viral infections and bacterial infections from others in clinical rescue, the average AUC of these two categories directly guides the choice of classifier (Table 3, Fig. 4). Moreover, accuracy, sensitivity, and specificity were used to evaluate models in the training, test and validation sets (Table 2, Fig. 4D).

**Table 2.** ACC, sensitivity, and specificity of the four models.

		ACC			Sensitivity			Specificity		
Model	Pathogen	Train	Test	Validation	Train	Test	Validation	Train	Test	Validation
CART	Bacterial	1.000	0.872	0.866	1.000	0.702	0.857	1.000	0.913	0.878
	Viral	1.000	0.842	0.866	1.000	0.858	0.626	1.000	0.823	0.944
	Noninfected	1.000	0.879	0.873	1.000	0.736	0.870	1.000	0.928	0.874
Random forest	Bacterial	1.000	0.730	0.571	1.000	0.313	0.378	1.000	0.831	0.815
	Viral	1.000	0.733	0.653	1.000	0.754	0.778	1.000	0.708	0.612
	Noninfected	1.000	0.884	0.891	1.000	0.775	0.793	1.000	0.922	0.915
SVM	Bacterial	0.927	0.896	0.959	0.817	0.735	0.963	0.954	0.935	0.954
	Viral	0.919	0.897	0.973	0.910	0.892	0.944	0.929	0.904	0.982
	Noninfected	0.948	0.928	0.959	0.930	0.887	0.897	0.955	0.942	0.975
Deep neural network	Bacterial	0.936	0.898	0.918	0.920	0.822	0.986	0.939	0.916	0.833
	Viral	0.927	0.876	0.973	0.958	0.927	0.959	0.892	0.813	0.977
	Noninfected	0.943	0.921	0.972	0.950	0.926	0.954	0.940	0.920	0.977

**Table 3.** AUC of the four models for the test set.

Model	Bacterial	Viral	Noninfected
Decision tree	0.808	0.840	0.832
Random forest	0.572	0.731	0.848
SVM	0.835	0.898	0.914
Neural network	0.954	0.956	0.975

### 2.5 Function enrichment

To explore the functions the identified gene pairs implemented, hypergeometric distribution was used for function enrichment of Gene Ontology terms (Gene Ontology Consortium 2021) and KEGG pathways (Kanehisa et al. 2021). *clusterProfiler* in R environment (Yu et al. 2012) was used for the evaluation.

## 3 Results

### 3.1 Assembling of large-scale host transcriptome data

To train a high-performing model, 3203 samples across 16 cohorts were retrieved from the Gene Expression Omnibus (GEO) database (Edgar et al. 2002) (Supplementary Fig. S1). We excluded patients that were not infected but with other diseases (108 with sarcoid, 16 with cancer), patients with unknown pathogens (360), and 39 coinfected patients. After the elimination, 2533 samples were established as the discovery set quantifying the gene expression of the whole blood of patients that were bacterial infected (503 samples), viral infected (1365 samples), or noninfected (665 samples) (Table 1). We randomly selected 70% of the samples for model training to extract the GPSs and train the antibiotics decision model (Fig. 1A), resulting in a training set of 356 bacterial, 947 viral, and 470 noninfected samples as well as a test set (30%) of 147 bacterial, 418 viral, and 195 noninfected ones for performance evaluation and model determination. Furthermore, to validate model generalizability, we applied 147 samples from two independent cohorts (GSE21802 and GSE57065) for external validation (Supplementary Fig. S1).

To conduct a large-scale host transcriptome analysis, individualized Pairwise Analysis of Gene Expression (iPAGE) was used to integrate the samples across multiple cohorts, which is a sophisticated strategy to remove batch effect and extract the common information from different cohorts based on the relative expression changes of gene pairs. Although two genes have different orders of magnitude in expression, their relative expression in an identical individual is robust to the technical variation within a cohort and batch effect among different cohorts. Thus, we hypothesized that gene pairs with a consistent relative expression change between two groups would most likely demonstrate the expression alterations. Using gene pairs, we integrated all the samples of the training set and screened out gene pairs with pronounced reversal on relative expression between a positive and a negative group to construct GPSs (see Section 2 and Fig. 2A). Since normalizations and other numerical transformations affected little on the relative expression of genes, these cohorts were not preprocessed and only the raw data were used.

### 3.2 Development of gene pair signatures

Since 7383 genes were detected by the samples, over 50 million gene pair would be calculated if exhaustive comparison was conducted. Directly measuring thousands of gene pairs and performing millions of combinations would lead to enormous time complexity and dimensional disasters that severely affected the efficiency in distinguishing pathogens. To this end, we performed pair extraction separately in each biological pathway, i.e. MSigDB’s curated gene sets (Subramanian et al. 2005) to speed up the detection, because the infection is more likely to cause changes of gene relative expression in specific pathways (Fig. 2B). Subsequently, we used Fisher’s exact test to identify the gene pairs with the most significant difference in reverse expression between the positive and negative groups (Fig. 2A). Bonferroni correction was used to control the false discovery rate at a significance level of 10–10. Finally, 40 477, 30 954, and 102 393 gene pairs were screened out as differentially expressed for noninfected, bacterial, and viral categories, respectively.

Using LASSO, the selected gene pairs were further refined by modeling them in the training set for each infection category (Fig. 2A, see Section 2). LASSO penalized the redundant and correlated gene pairs to screen out the minimum number of gene pairs. Ultimately, we obtained 54 unique gene pairs, 21, 24, and 11 for bacterial, viral, and noninfected, respectively (Supplementary Fig. S2). Two gene pairs, *CTSD-MCOLN1* and *HERC2-UBE2C*, were identified as markers in both the viral and noninfected categories but with opposite reversals (Supplementary Fig. S3). The weighted combination of these gene pairs functions as indicators for each type of infection and we referred them to bacterial GPS (bGPS), viral GPS (vGPS), and noninfected GPS (nGPS) (Fig. 2C–E).

### 3.3 Construction of multiclass neural network

**Figure 3:**
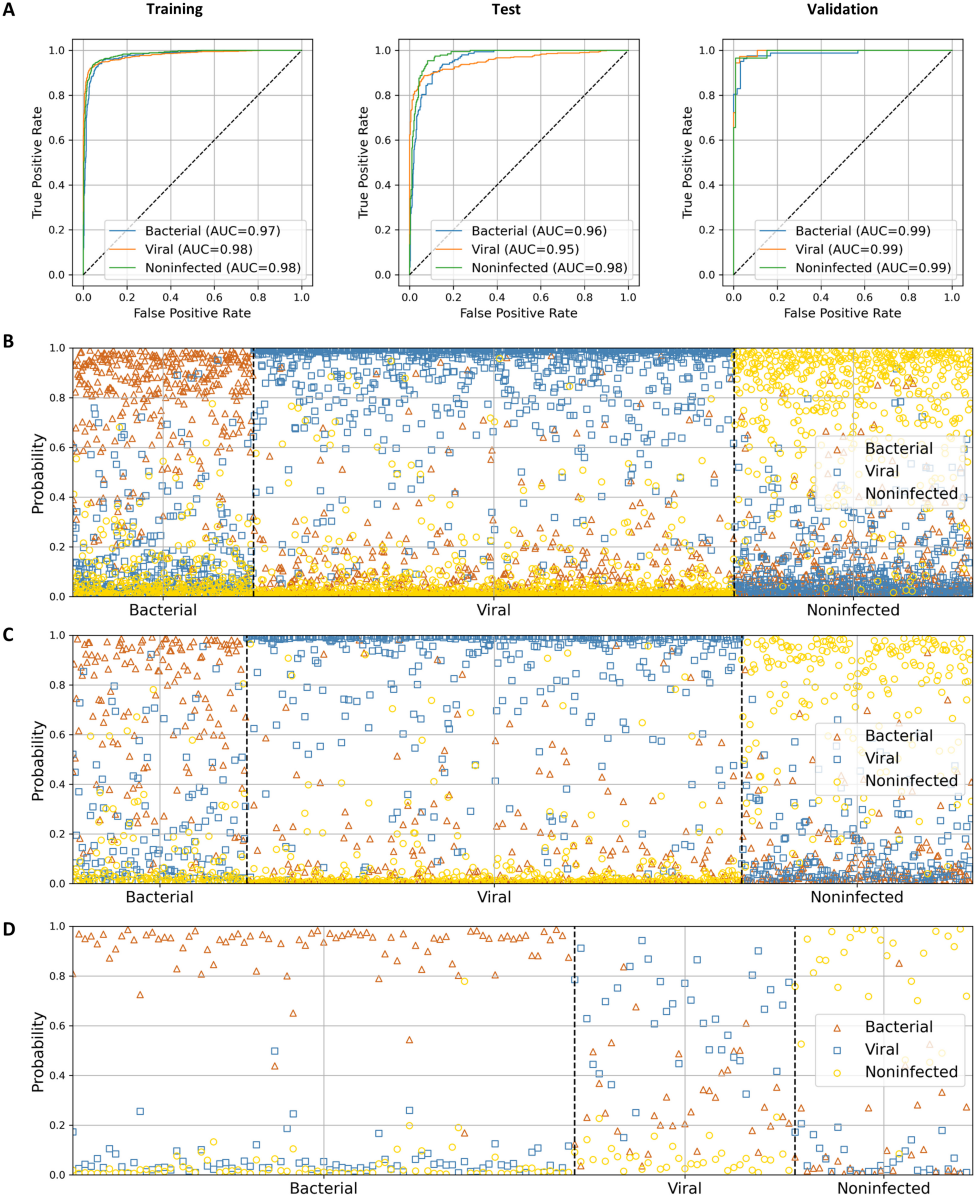
Evaluation of the bvnGPS model. (A) ROC curves of the model for the training set, test set, and validation set. The horizontal axis represents the three types of patient while the vertical axis represents the highest probabilities of the patient assigned to bacterial infection (triangle), viral (square), or no infection (circle) for the training set (B), test set (C), and validation set (D)

Multiclass neural network with pretrained features was employed to assemble the three GPSs into an antibiotics decision model (bvnGPS) to distinguish patients from bacterial infection, viral infection, and noninfection. In bvnGPS, the structure of model consists of a feature generation module and a bvn three classification module. In the feature generation module, binary labels are used to train a single class of features. In the classification module, bvnGPS concatenates the features of bacteria, virus, and noninfected as well as classifies them into three categories. Specifically, the best-performed hyperparameter combination is that the feature generation module has 64 hidden units and the bvn three classification module contains no hidden layers (Fig. 1B). Trained in 38 epochs, the model yielded a sensitivity of 71.5% and a specificity of 94.9% for bacterial infection while a sensitivity of 88.9% and a specificity of 90.7% for viral infection in the test set of 760 samples (Table 2). The model differentiated specific infection type from others for both bacterial infection (ACC = 0.904) and viral infection (ACC = 0.897) in the test set (Table 2). It demonstrated AUCs of 0.974 [95% confidence interval (CI), 0.968 to 0.980] for bacterial infection, 0.980 (95% CI, 0.973 to 0.987) for viral infection, and 0.984 (95% CI, 0.980 to 0.988) for noninfected in the training set, and AUCs of 0.953 (95% CI, 0.948 to 0.958) for bacterial infection, 0.956 (95% CI, 0.951 to 0.961) for viral infection, and 0.975 (95% CI, 0.971 to 0.979) for noninfected in the test set (Fig. 3, Table 3).

**Figure 4:**
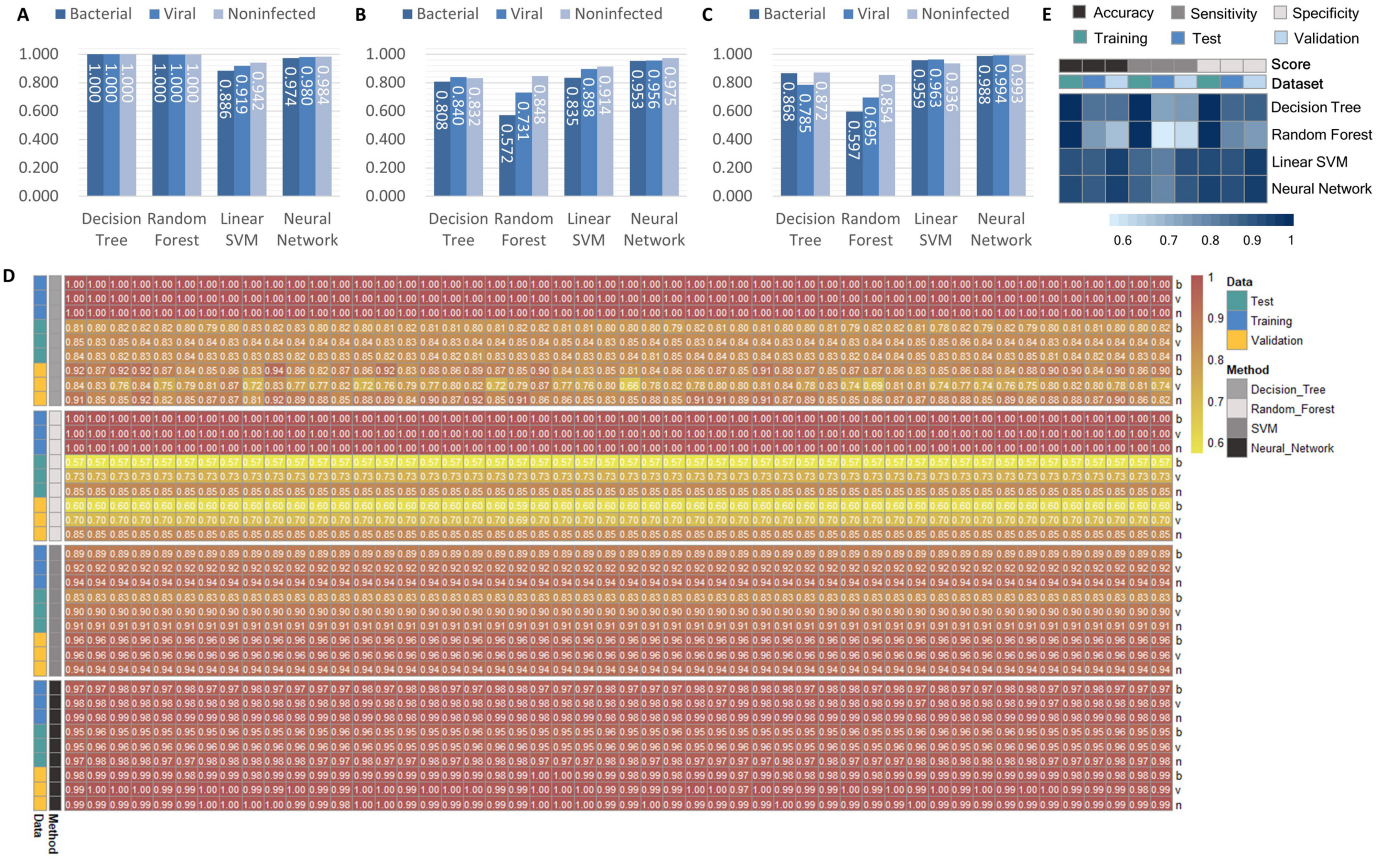
Comparison of multiclass machine learning models for the assembling of gene pairs. (A–C) AUC of different models for the training, test, and validation sets. (D) Heatmaps of classifier performance metrics (AUROC) from red (high) to yellow (low) for distinguishing between infection types. b, v, and n represent bacterial infection, viral infection, and noninfected, respectively. (E) Heatmaps of micro-averaged accuracy, sensitivity, and specificity, from dark (high) to light blue (low)

We also implemented three other multiclass machine learning models for comparison (Fig. 1), i.e. DT, RF, and SVM. We trained these models on the training set 50 times with different random parameter initialization and reported the average performance. In the test dataset, the 50 times averaged AUCs of the bacterial infection were around 0.808, 0.572, and 0.835, and AUCs of the viral infection were around 0.840, 0.731, and 0.898 for DT, RF, and SVM, respectively (Fig. 4B, Table 3). In summary, the neural network had better generalization ability than DT and RF that may overfit the training set (Fig. 4A, Supplementary Fig. S4). Compared to SVM with linear kernel, neural network achieved better results in AUC (0.953 versus 0.835 for bacterial, 0.956 versus 0.898 for viral, and 0.975 versus 0.914 for noninfected) (Fig. 4B, Table 3).

### 3.4 Validation in independent datasets

To verify the performance of bvnGPS, we combined GSE21802 and GSE57065 containing either viral infection or bacterial infection as the external validation set (Fig. 1). The bvnGPS had AUCs of 0.988 (95% CI, 0.978 to 0.998), 0.994 (95% CI, 0.984 to 1.000), and 0.993 (95% CI, 0.988 to 0.998) for bacterial, viral, and noninfected, respectively, in the validation dataset (Fig. 4C). Performance of the models with 50 random parameters was visualized in a heatmap (Fig. 4D), indicating that bvnGPS using multiclass neural network generally outperforms DT, RF, and SVM in both the test set and the validation set. Furthermore, bvnGPS demonstrated superior sensitivity and specificity for classification than the other three models (Table 2).

Furthermore, bvnGPS achieved high specificity of 0.953 and 0.990 for the noninfected samples in the test set and validation set, respectively, which indicated the infected samples could be identified with high confidence. With the ‘just in case’ strategy, antibiotic prescription on infected patients based on bvnGPS is able to lower missed diagnosis of bacterial infection. After micro averaging, bvnGPS achieved the highest accuracy and sensitivity on the test set and validation set (Fig. 4E), although its specificity was second to SVM. Collectively, bvnGPS using gene pairs and neural network achieved an outstanding performance, suggesting that the pattern of gene pairs could provide reliable discriminatory markers for the detection of pathogens.

### 3.5 Functional analysis

**Figure 5:**
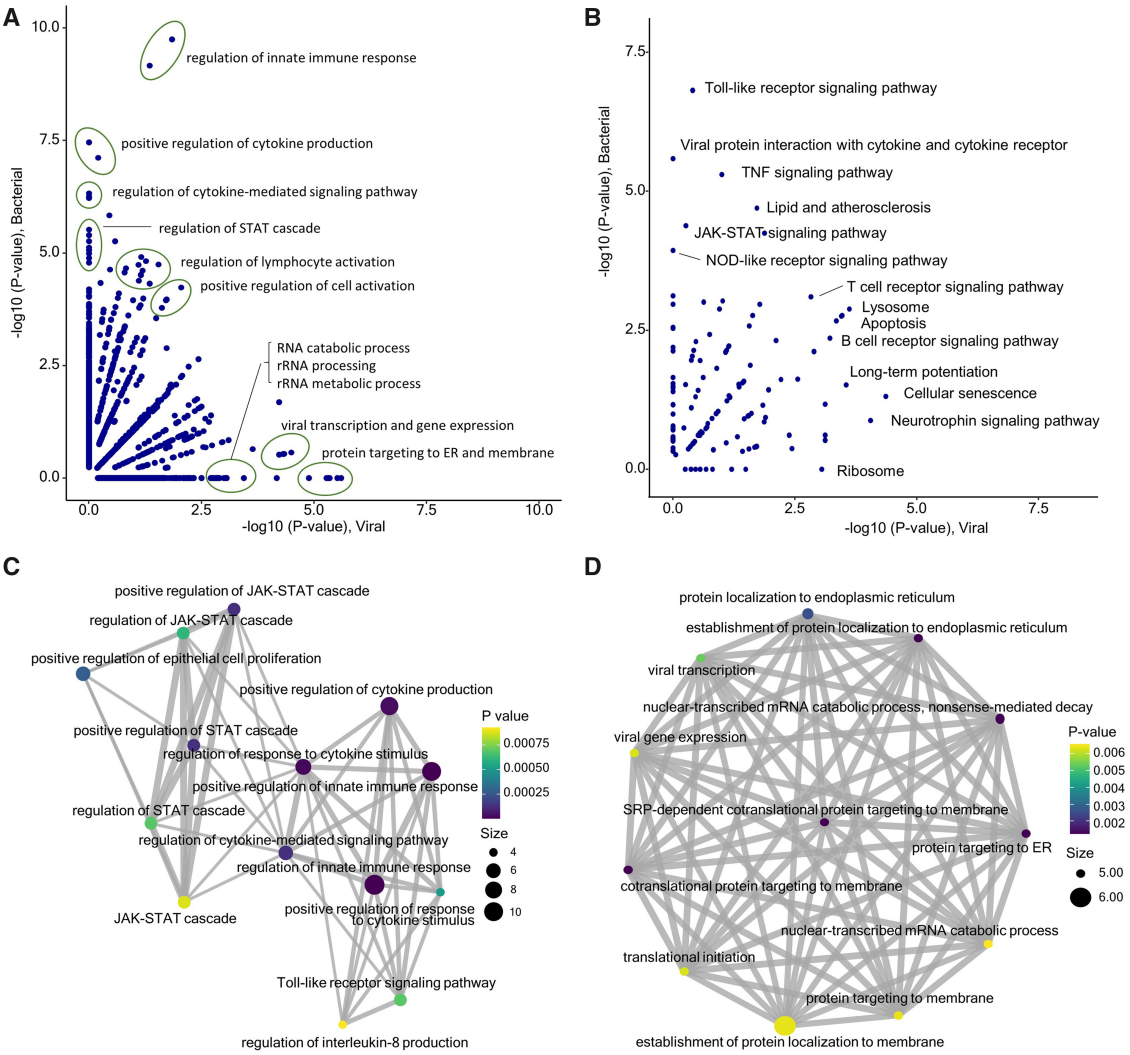
Functional analysis of the gene pairs in bGPS and vGPS. Scatterplots of the significance level from function enrichment analysis between bGPS gene pairs and bGPS gene pairs for Gene Ontology (A) and KEGG (B). Network plot of the enriched biological processed for bacterial infection (C) and viral infection (D), respectively. The size of the vertex corresponds to the number of genes in the GO term, while the color represents statistical significance. Edge indicates the proportion of genes shared by GO terms

We further explored the significant biological functions and pathways where the bGPS and vGPS gene pairs are involved (Fig. 5). Gene Ontology (Gene Ontology Consortium 2021) function analysis illustrated the bGPS and vGPS genes are overrepresented in distinct biological pathways. Specifically (Fig. 5A, top left), the gene pairs in bGPS are significantly enriched in the biological processes of signal transducer and activator of transcription (*STAT*) and Janus kinase-signal transducer and activator of transcription (*JAK-STAT*) cascade and immune-related functions including regulation of innate immune response, regulation of cytokine production, regulation of lymphocyte activation, and regulation of cytokine mediated signaling pathway (Fig. 5C). On the other hand, at the bottom right of Fig. 5A, the vGPS are involved in pathways of viral gene transcription and targeting, such as viral transcription, protein targeting to membrane, protein targeting to endoplasmic reticulum (ER), and protein localization to ER (Fig. 5D). On the other hand, vGPS are related to physiological processes of cells, such as long-term potentiation, cellular senescence, and ribosome which are associate with the proliferation of viruses (Fig. 5B). Overall, the gene pairs for bacterial infection and viral infection are implemented in distinct functions, not limited to the biological processes, but also the molecular functions and cellular components (Supplementary Fig. S5).

Several previous studies have reported the genes in vGPS and bGPS are highly relevant to bacterial and viral infection. For gene pair *CD1A* and *CD1C* in vGPS, both were revealed to bind self and non-self-lipid and glycolipid antigens, presenting them to T-cell receptors on natural killer T-cells (Sugita et al. 2000; Vincent et al. 2005). Moreover, for bGPS gene pair *AIM2* and *ZBP1*, a recent research has shown that *AIM2* is part of the inflammasome and contributes to the defense against bacterial and viral DNA (Schroder and Tschopp 2010).

## 4 Discussion

We collected and integrated the bacterial and viral datasets by our proposed model iPAGE to enlarge the sample size for a large-scale host transcriptomic analysis (Zheng et al. 2021). Bacterial, viral, and noninfected GPSs were separately extracted and then assembled in an antibiotics decision model (bvnGPS) using a multiclass neural network to determine the infection types. With the high AUC, accuracy, sensitivity, and specificity of bvnGPS, we demonstrated it outperformed other machine learning and state-of-the-art models, i.e. the IMX-BVN-1 model from Stanford ICU (Mayhew et al. 2020). More specifically, unlike Sweeney et al. (2016), we retained the 90 noninfectious illness in GSE63990 as the control with other healthy samples. As previously described by Tsalik et al. (2016), noninfectious illnesses are more likely to present in clinical practice and be a potential source of diagnostic error and misdiagnosed by many classifiers for they could not be a poor substitute with healthy samples. By contrast, our results on the test set and validation set demonstrated the robustness of bvnGPS to the noninfectious illnesses (Table 2).

In previous studies, models were usually trained and validated on an individual cohort (Almansa et al. 2012; Suarez et al. 2015; Burnham et al. 2017), hindering the utilization of the neural network model with more parameters and generalization to clinical practice. However, assembling various data sets is not easy work, since batch effect is an integral factor in precluding model performance with different background measurements (Fu et al. 2022; Li et al. 2022). Generally, conormalization methods are indispensable for integrating multiple cohorts from different laboratories or platforms to improve model robustness. Sweeney et al. (2016) proposed COCONUT derived from ComBat (Johnson et al. 2007) referring healthy samples assumed in the same background distribution as references and normalized expression of all patients to a relatively comparable magnitude. However, the above biomarker screening method does not fit datasets without control samples, such as GSE68310, a cohort with only viral and bacterial infections. Besides, normalization methods process gene expression value based on disparate assumptions, and only datasets that meet the corresponding assumptions can be appropriately preprocessed (Hubbell et al. 2002; Irizarry et al. 2003; Cheng et al. 2016a,b; Liu et al. 2019).

For GPS assembling, neural networks achieved better performance than DT, RF, and SVM with an average AUCs as high as 0.991 (0.988 for bacteria-vs.-other, 0.994 for virus-versus-other, and 0.993 for uninfected-versus-other) in the validation set (Fig. 4C). The superior performance of bvnGPS arose from the advantages of iPAGE and neural network: (i) iPAGE assembles multiple cohorts and purifies the most reliable information; and (ii) neural network is compatible with the numerous low-density information in relative expression for a large number of parameters.

Although discrimination of bacterial and viral infections helps a lot in guiding a clinician to use antibiotics appropriately, accurate identification of specific pathogens would be a crucial requisite for optimally targeted antimicrobial treatment. However, pathogen prediction is a multilabel classification problem, which is mainly challenged by the limited sample size. In this work, we have performed a systematic search for host transcriptome datasets and collected around 3000 samples, but the sample size for each is yet insufficient to build an accurate model for specific pathogens. In our future work, we will build a more precise model sensitive to specific pathogens once sufficient data is available.

In conclusion, we constructed three GPSs for different infection types based on a large-scale integrative host transcriptomic data. Multiclass neural network was utilized to assemble these GPSs and developed the final model bvnGPS, which achieved a promising performance in the test set and external validation set. bvnGPS demonstrated its potential in reducing mortality and antibiotics overuse in clinical practice. Prospective clinical validation is warranted before this prediction model is used for patient care. This method also verified the reliability and robustness of relative expression and provided inspiration for further omics studies.

## Supplementary Material

btad109_Supplementary_Data

## Data Availability

The data underlying this article are available in the GEO database, at https://www.ncbi.nlm.nih.gov/geo/. Data used for training and testing are available in Table 1.
