# Supplementary material for: bvnGPS: a generalizable diagnostic model for acute bacterial and viral infection using integrative host transcriptomics and pretrained neural networks
Source: Bioinformatics. 2023 Mar 1;39(3):btad109. doi: 10.1093/bioinformatics/btad109 (PMC9997702; doi:10.1093/bioinformatics/btad109)
Supplement: btad109_Supplementary_Data [file btad109_supplementary_data.zip › Supplementary.docx]

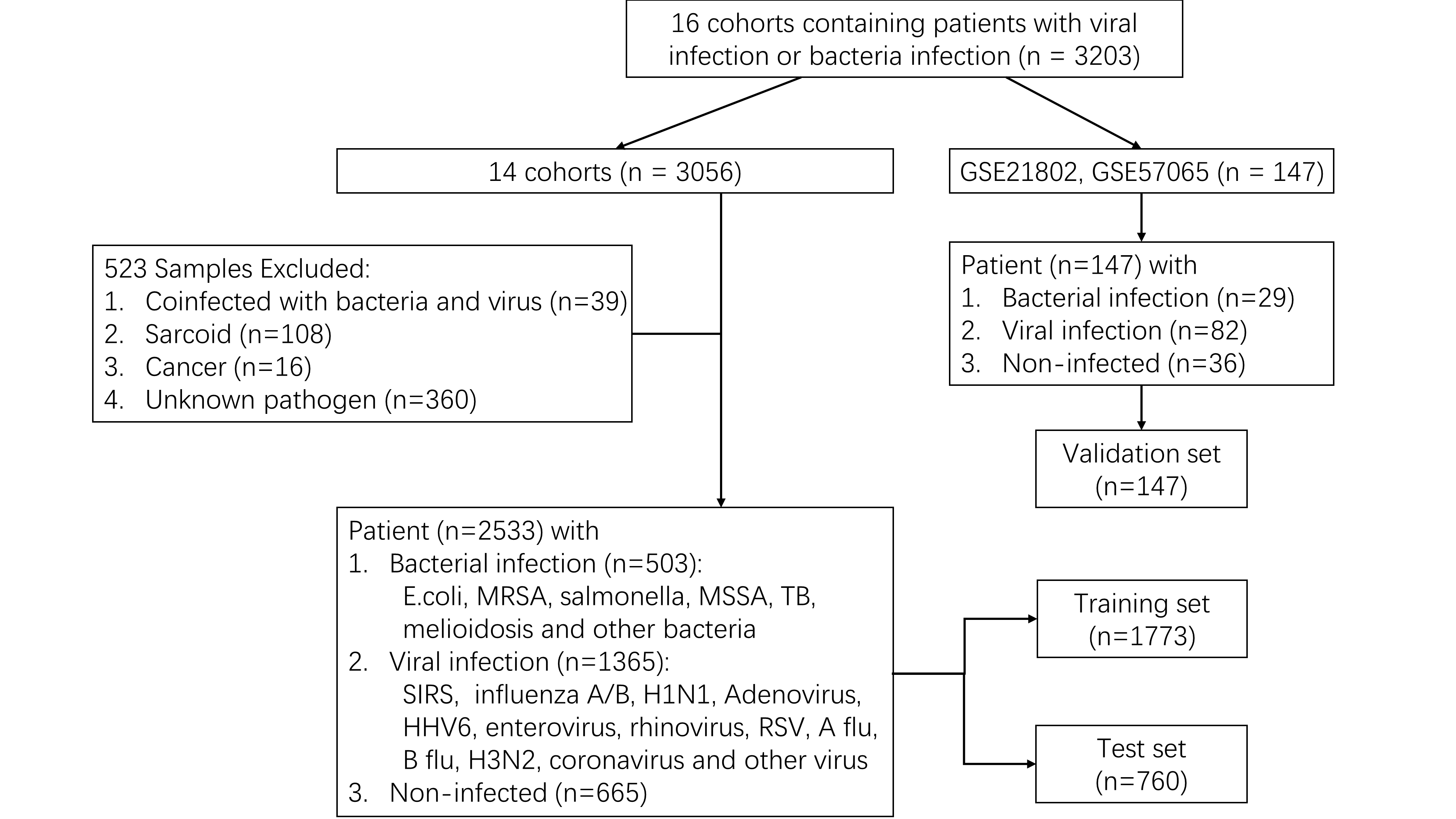


**Figure S1.** Flowchart of cohorts and samples according to inclusion and exclusion criteria.


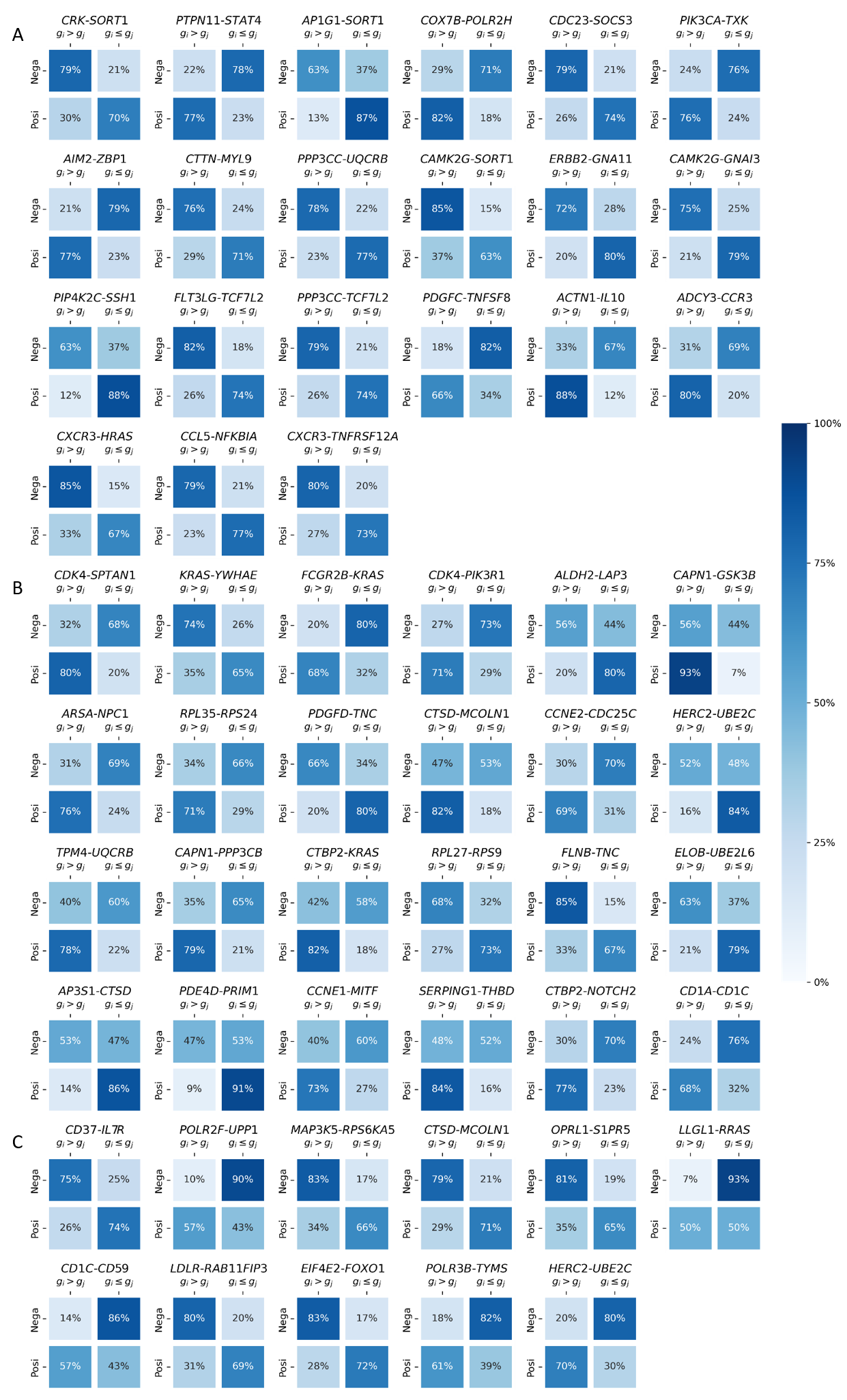


**Figure S2.** Contingency table of the identified gene pairs in gene pairs for bacterial (**A**), viral (**B**), and noninfected category (**C**), respectively.


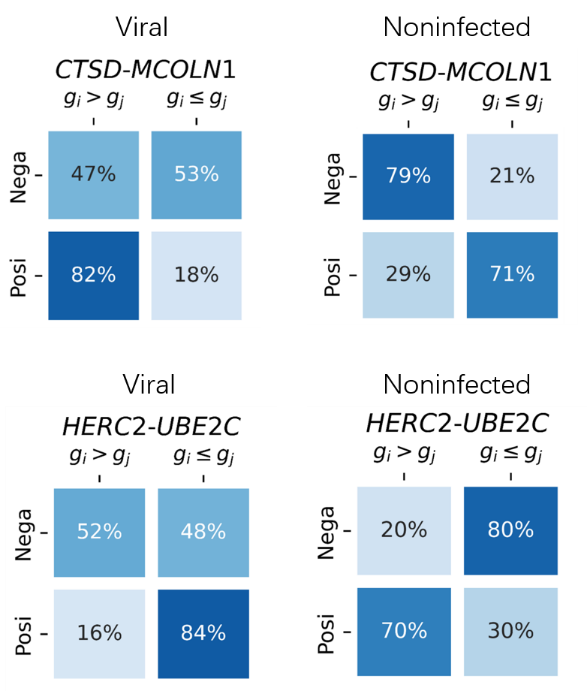


**Figure S3**. Contingency table for gene pairs *CTSD*-*MCOLN1* and *HERC2*-*UBE2C* when viral infection or noninfected group is defined as the positive group.


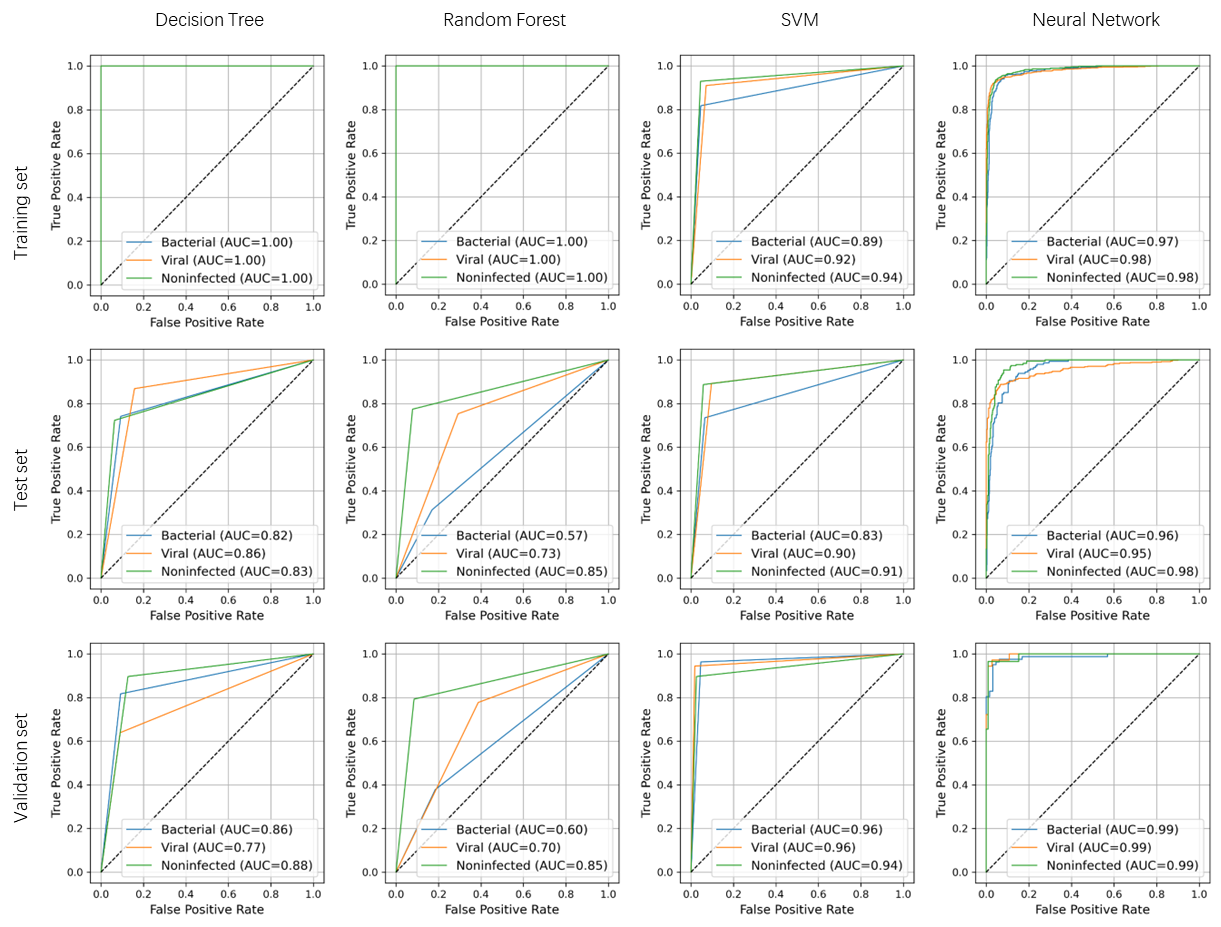


**Figure S4.** ROC curves of the prediction model for the training set, test set, and validation set using DT, RF, SVM and NN.


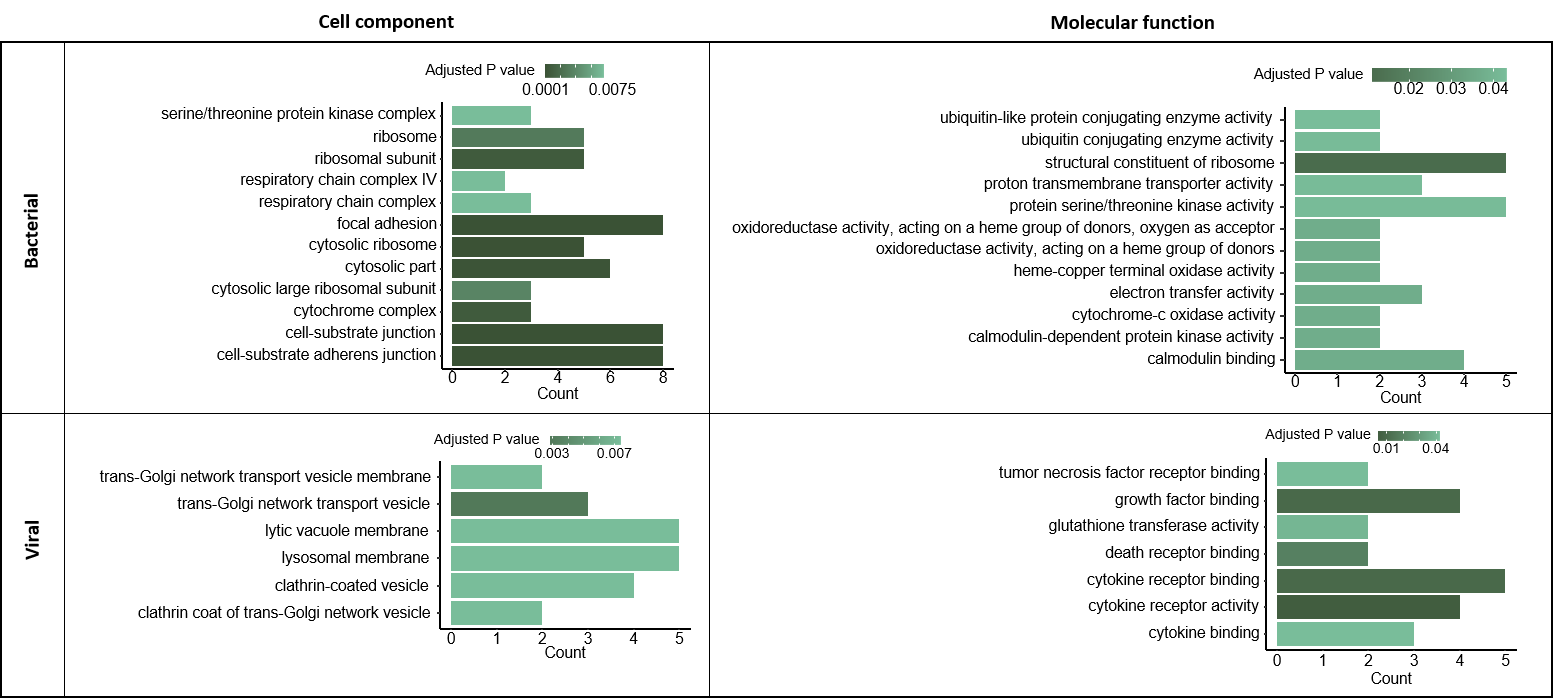


**Figure S5.** Function enrichment analysis of the gene pairs in bGPS and vGPS.


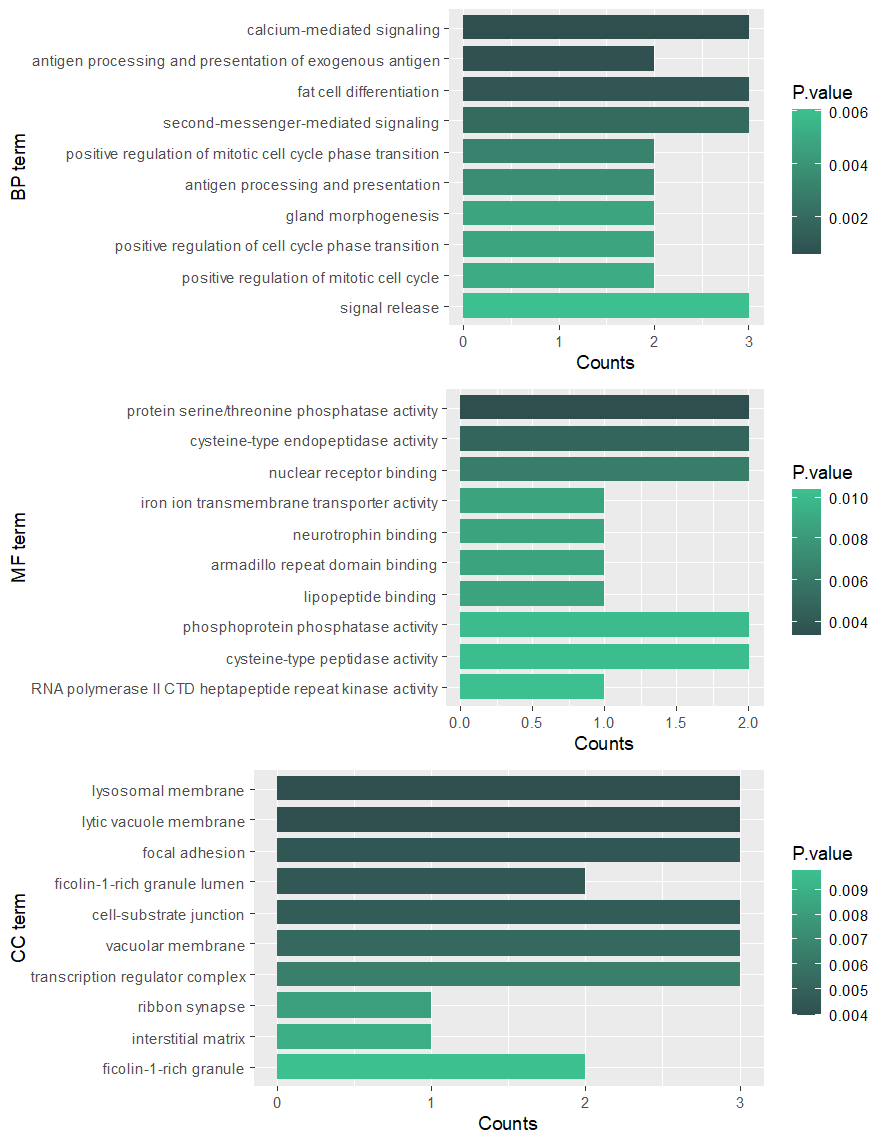


**Figure S6.** Function enrichment analysis of the genes appearing in multiple gene pairs of bvnGPS.
